# Dual-Polarized Metal Vivaldi Array Using Independent Structural Elements

**DOI:** 10.3390/s24020315

**Published:** 2024-01-05

**Authors:** Bo Chen, Wei Wang, Juan Liu, Jianmin Ji

**Affiliations:** Beijing Institute of Remote Sensing Equipment, Beijing 100854, China

**Keywords:** Vivaldi, dual-polarized, phased array

## Abstract

In this letter, a dual-polarized metal Vivaldi phased array antenna composed of independent structural elements is proposed, covering 6–18 GHz. By designing Vivaldi elements with a flexible and complementary structure, arrays of arbitrary shapes and scales can be constructed. The resonance caused by structural discontinuity is critically studied and eliminated to ensure good performance across the entire band. The antenna elements are fed by 50-Ohm SSMP connectors and manufactured from 2A12 aluminum alloy. An array prototype consisting of 8 × 8 dual-polarized metal has been fabricated and tested with active transmit/receive (T/R) modules to demonstrate the design concept. The array exhibits excellent beam-scanning characteristics in both the E-plane and H-plane, within the scanning range without grating lobes, which shows good agreement with the simulated results. The measured gain results are within the range of 15.2 to 24.8 dBi, and the aperture efficiencies are greater than 91% in the entire operating band. The wideband antenna technology involved in this study can effectively help increase the capacity of communication systems and meets the intentions of the Special Issue.

## 1. Introduction

Wideband or multi-band antennas are very valuable for improving the performance of wireless communication systems, especially for multi-functional integrated communication systems [1,2,3,4,5]. Since Gibson first proposed the Vivaldi antenna in 1979, it has been demonstrated to be a critical technology for providing a wide operating bandwidth and has been widely utilized in various systems [6]. The increasing demand in radar and electronic countermeasures for broadband and ultra-wideband phased arrays has stimulated comprehensive investigations into Vivaldi antenna designs in the past few years [7,8,9,10,11]. Generally, conventional Vivaldi antennas were designed and manufactured based on a printed circuit board (PCB), which has the advantages of high processing precision, good end-fire radiation, and low cost [12,13,14,15,16,17]. Due to the characteristics of high strength, high temperature and power resistance, some designs for metal Vivaldi arrays have been presented in the past decade [18,19,20,21,22,23,24,25,26]. In [18], Kindt and Pickles presented a design for an all-metal Vivaldi array element with an operational bandwidth of 12:1 for broadside scanning and 8:1 bandwidth at a 45-degree scan in all planes. The elements were constructed entirely of 6061 aluminum using wire EDM cutting technology and fed via “fuzz-button” connectors. In [21], according to Kindt’s work, a 2–18 GHz dual-polarized Vivaldi antenna array for airborne radar was presented, and the array was able to scan up to 30°. In [24], a type of 3D additively manufactured metallic Vivaldi array was reported, exhibiting the features of cheap and rapid manufacturing. In summary, excellent structural stability and radiation performance can make the metal Vivaldi antenna suitable for further applications. However, the arrays proposed in the above papers are all fabricated in one piece. This design approach is not flexible enough to constitute large-scale arrays, and it is not convenient for T/R modules to connect. It is more advantageous to form the array by using elements or small subarrays with an independent structure, which can be easily inserted or removed from the aperture. It is, therefore, necessary to investigate and develop a metal Vivaldi antenna array composed of independent structural elements to support ultra-wideband radar, communications, or other electronic systems.

In this letter, we will focus on improving the structural adaptability compared to existing metal Vivaldi designs. A novel metal dual-polarized Vivaldi antenna element with a complementary structure and an 8 × 8 finite phased array prototype is presented. It exhibits a 3:1 bandwidth covering 6–18 GHz and good scanning characteristics. During our research, we found that the structural discontinuity of the array brought by elements would cause the array to resonate at certain frequencies, thus leading to a deterioration in the array performance. A similar phenomenon of wideband performance deterioration in the PCB-based Vivaldi arrays caused by the separation of the adjacent elements has been found in previous studies, such as [7,27]. To overcome this problem, a method of eliminating the resonance was specifically studied and verified and has become an important feature of our work. Commercial SSMP connectors soldered to the elements are used as excitation ports, which not only reduce the height of the entire antenna, but also make it easier to integrate with T/R modules to form an active phased array. Furthermore, the manufacturing and assembly processes are discussed. The proposed Vivaldi element structure features several advantages compared to those in previous studies.

## 2. Antenna Element Design and Analysis

### 2.1. Antenna Element Design

**Figure 1:**
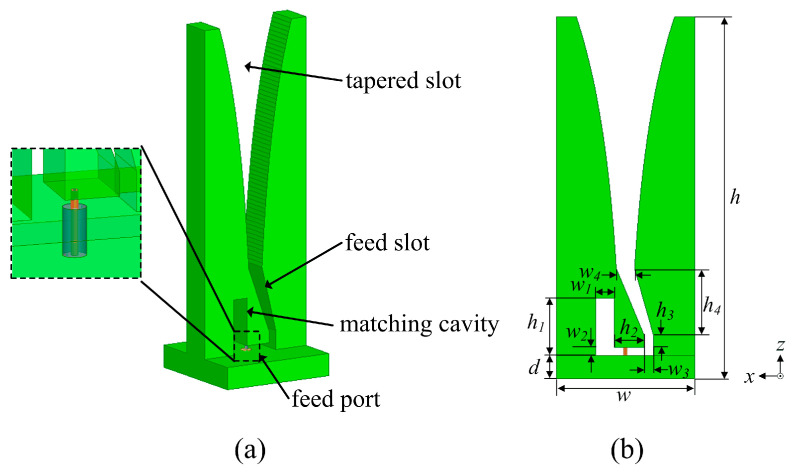
Geometry of the proposed Vivaldi element with key parameters. (**a**) The 3D view. (**b**) The front view.

The basic structure used in this study was presented in [13], and has been referenced and modified several times in recent years. Figure 1 shows the profile of the proposed metal Vivaldi element, including its 3D and front views, along with key parameters. The element consists of a tapered slot, feed slot, matching cavity, and 50 Ω coaxial feed port. The coordinate system is also identified in the figure. It is noted that the *xz*-plane and *yz*-plane are, respectively, referred to as the E-plane and H-plane of the element.

The antenna element lattice spacing for each polarization is set as 10.5 mm × 10.5 mm, which achieves the ability to scan to 45° at 6–16.7 GHz and to 35° at 16.7–18 GHz in both the E- and H-planes, respectively, according to the condition that no grate lobe appears in the visible space [28]; that is,

(1) $$\frac{d}{\lambda }<{\left(1+\sin\theta \right)}^{-1}$$

where *d* is the distance between adjacent elements, *λ* is the wavelength in the free space of the operating frequency, and *θ* is the scanning angle.

The exponential profile curve employed in this design can be described by the following equation:

(2) $$x=c_{1}\times e^{Rz}+c_{2}$$

where *R* is the opening rate, and *c*_1_ and *c*_2_ are determined by the coordinates of the first and last points of the exponential curve.

The element is simulated and optimized via ANSYS HFSS to obtain the desirable performance covering 6–18 GHz. The periodic boundary condition is used to analyze the performance of the antenna element in an infinite array environment. The minimum operating frequency of the embedded elements is determined by the antenna height, *h*, and the array size. As *h* increases, the minimum operating frequency decreases. The exponential opening rate of the antenna mainly influences the impedance matching performance of the lower band. The final dimensions of the proposed antenna are listed in Table 1.

**Figure 2:**
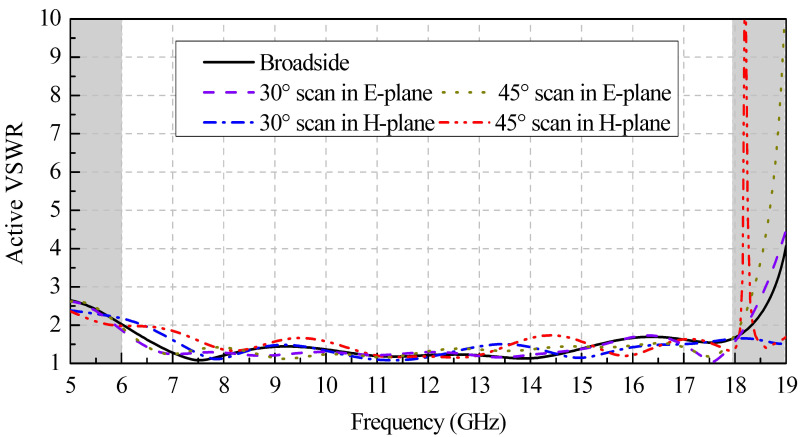
Simulated infinite active VSWR performance of the Vivaldi element at broadside, 30°, and 45° scans in the E- and H-planes.

Figure 2 shows simulated active VSWR results for the proposed design in an infinite array with different scanning angles in the E- and H-planes, respectively. It can be found that the broadside VSWR is less than 2.1 across the frequency band of 6–18 GHz. When scanning in the E- and H-planes, the active VSWR is less than 2.1 and less than 2.3 for all scanning angles to 45° over the entire band, respectively. No scanning blindness occurs for a 45° scan within the operating band.

### 2.2. Manufacturing Factor Analysis

**Figure 3:**
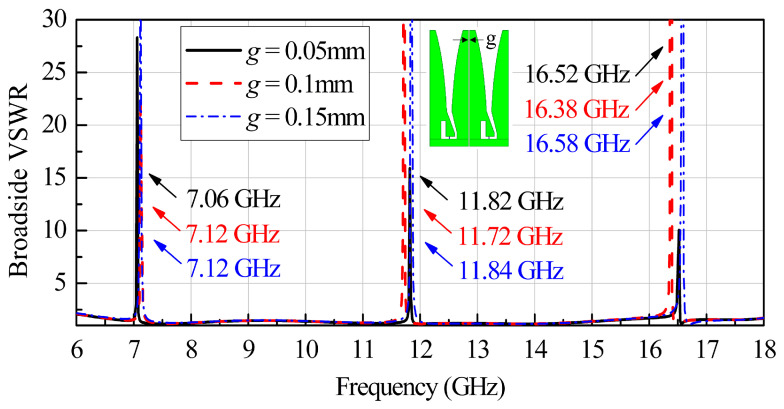
Simulated infinite broadside VSWR performance for different air gap widths *g*.

In the machining process, it is impossible to produce everything to an exact size. In order to ensure that each element can be smoothly assembled into an array on the substrate, it is necessary to limit the size L to a minus tolerance range during machining. Therefore, unlike the overall processing used in Kindt’s work, a perfect contact between two adjacent elements is difficult to achieve for this standalone mounting. As a result, there must be an air gap between two adjacent antenna elements. The width of the air gap is defined as *g*. To investigate the influence of the air gap width on the antenna performance, simulations taking into account air gaps are carried out for different values of *g*. Figure 3 plots a comparison of the simulated broadside VSWR curves when *g* = 0.05 mm, 0.10 mm, and 0.15 mm. It can be observed from Figure 3 that the air gap between two adjacent co-polarized antenna elements can cause severe deterioration in VSWR at some singular frequencies. For the case of *g* = 0.05 mm, the singularities occur at the frequencies of 7.06 GHz, 11.82 GHz, and 16.52 GHz. For the case of *g* = 0.10 mm, the singularities occur at the frequencies of 7.12 GHz, 11.72 GHz, and 16.38 GHz. For the case of *g* = 0.15 mm, the singularities occur at the frequencies of 7.12 GHz, 11.84 GHz, and 16.58 GHz. In addition, when g increases from 0.05 mm to 0.15 mm, the resonant frequencies shift slightly.

**Figure 4:**
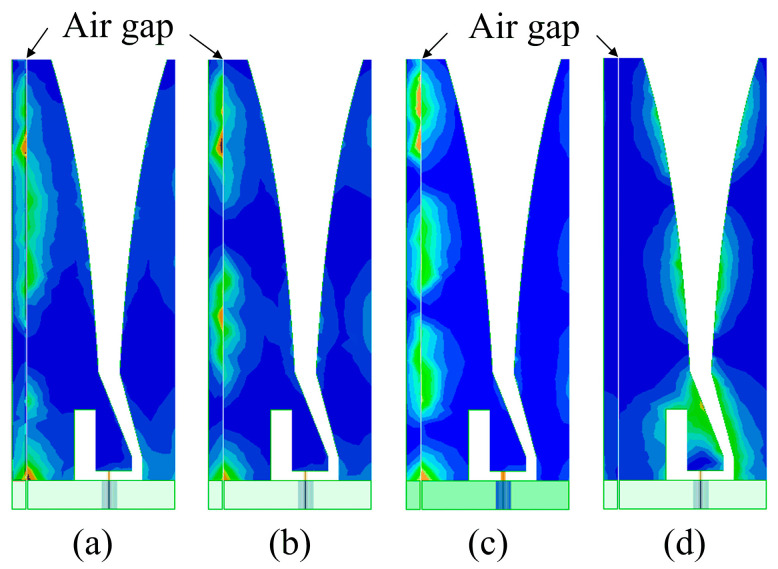
Surface current distributions on the front of the element at three singular frequencies and a normal one, when *g* = 0.1 mm: (**a**) 7.12 GHz, (**b**) 11.72 GHz, (**c**) 16.38 GHz, and (**d**) 14 GHz.

Taking *g* = 0.10 mm as an example, the surface current distributions on the front of the element at singular frequencies and a normal one (14 GHz) are presented in Figure 4 to help us further understand the behavior of resonance effects. For frequency singularities, the current distribution around the air gap is obviously stronger than that along the tapered slot, which will lead to severe radiation power cancellation. However, the current mainly exists along the tapered slots when the antenna works at normal frequencies, and this suggests that it benefits in terms of radiating electromagnetic energy into space.

**Figure 5:**
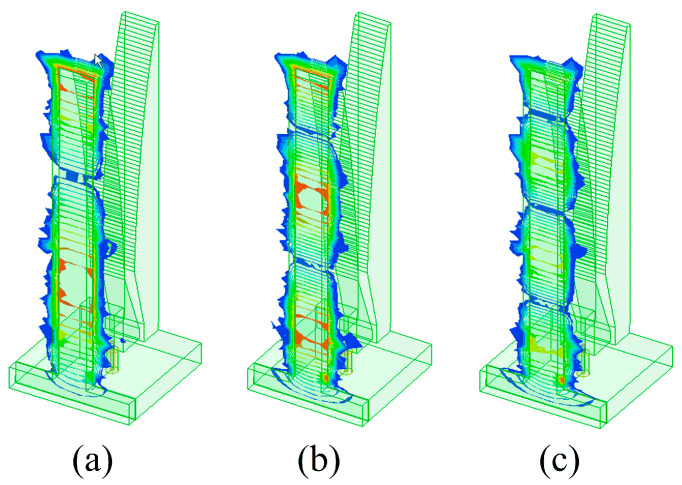
Electric field distributions in the air gap at singular frequencies when *g* = 0.1 mm: (**a**) 7.12 GHz, (**b**) 11.72 GHz, and (**c**) 16.38 GHz.

Furthermore, the simulations of the electric field in the air gap and its surrounding space are performed, as shown in Figure 5. The electric field distribution is very similar to that of a rectangular resonant cavity. The difference is that the air gap is a semi-open cavity. Combined with the standing wave numbers shown in Figure 5, the resonance modes can be viewed as TE_102_, TE_103_, and TE_104_ at the frequencies of 7.12 GHz, 11.72 GHz, and 16.38 GHz, respectively, according to [29].

### 2.3. Resonance-Suppression Method

**Figure 6:**
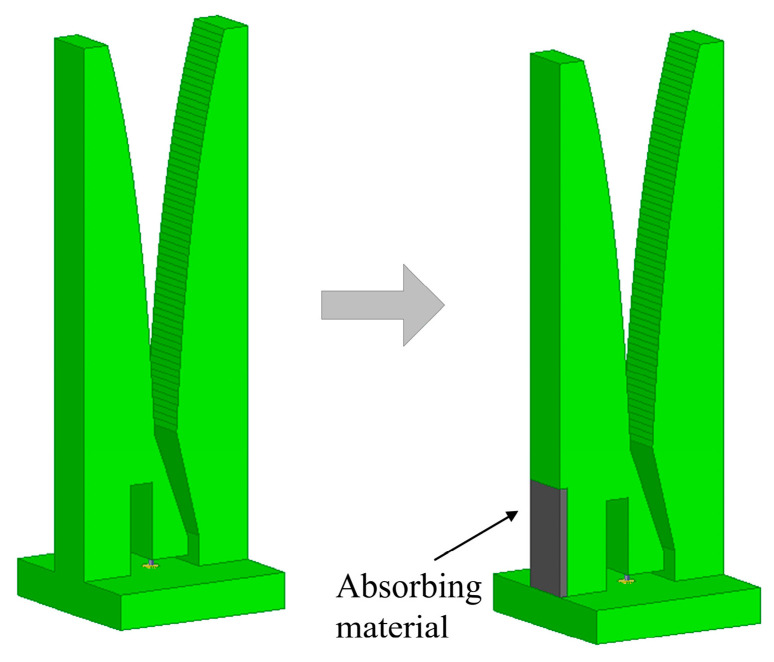
The improvement in the metal Vivaldi element.

Since the elements are independent, the air gap cannot be completely enclosed without receiving coupling from the feed port. Here, for the first time, we propose the introduction of an absorbing material into a metal Vivaldi element to suppress the formation of resonant fields without affecting the radiation performance, thereby improving the array performance in the operating band. Figure 6 displays the improvement in the metal Vivaldi element. A rectangular groove is processed on the side arm of the element, in which absorbing material is pasted. As for the absorbing material, we chose the Eccosorb BSR-1 with a standard thickness of 0.25 mm (0.010″) produced by Laird Technologies, which is designed for the frequency range from 6 to 35 GHz. Through simulation analysis, the dimension of the loaded absorbing material is set to 3 mm × 6 mm.

**Figure 7:**
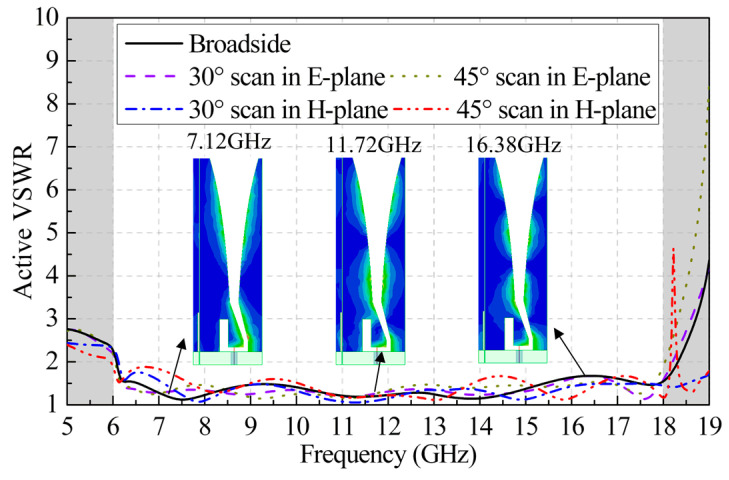
Simulated infinite broadside VSWR performance of the element with the absorbing material loaded when *g* = 0.1 mm, together with the current distributions at the original singular frequencies.

Figure 7 presents the simulated broadside and scanning active VSWRs of the Vivaldi element with the Eccosorb BSR-1 introduced to assess the effectiveness of the resonance-suppression method. It can be seen that the VSWR curves almost coincide with those in Figure 2. The simulated current distributions at 7.12 GHz, 11.72 GHz, and 16.38 GHz are also shown in Figure 7, respectively. It can be observed that the current flowing near the gap is significantly reduced by introducing the absorbing material compared to the current distribution shown in Figure 4 when there is no absorbing material between the two adjacent antenna elements.

As a result, due to the presence of the absorbing material, the impedance-matching characteristics are significantly improved: the resonant frequencies on VSWR curves have disappeared, which almost coincide with the results of the ideal element model without an air gap (that is, *g* = 0).

## 3. Manufacturing of Dual-Polarized Element

**Figure 8:**
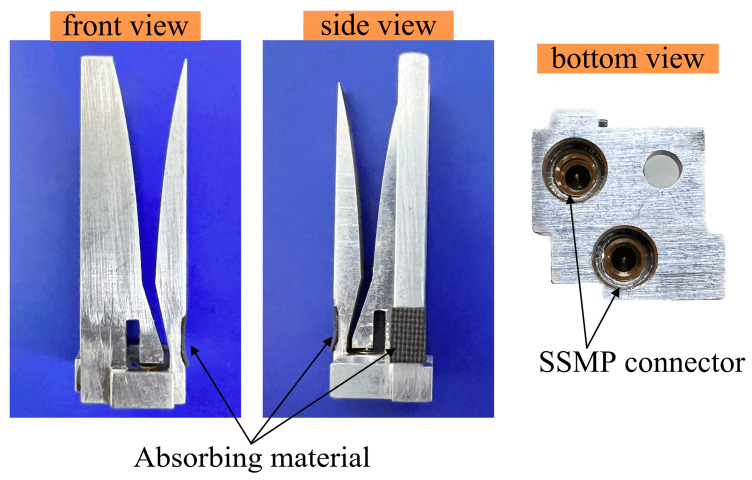
Photograph of the fabricated dual-polarized Vivaldi element including front, side, and bottom views.

Figure 8 displays the manufactured dual-polarized Vivaldi element based on the above design. The element is machined from 2A12 aluminum alloy using a high-precision wire-cutting technology. The two Vivaldi antennas are placed orthogonally to one another and joined at their side arms. A stepped-through hole with a 3 mm or 5 mm inner diameter is drilled into the ground of each element, through which an M2 screw can be inserted to help hold a metal plate together. The shape of the ground is specially designed to ensure that adjacent elements are complementary, so that arrays of any size can be assembled.

The dual-polarized element is fed at the bottom by two SSMP connectors with a characteristic impedance of 50 Ω. The integration procedures between the aluminum alloy element and the SSMP connectors are as follows. Firstly, a stepped hole and a blind hole are pre-machined on the element for connection with the outer and inner conductors of the SSMP connector, respectively. Secondly, the surfaces of the stepped and blind holes are evenly coated with conductive glue. Thirdly, the antennas and connectors are assembled and soldered together in a vacuum-brazing furnace. Finally, two pieces of absorbing material are pasted into the pre-machined rectangular grooves on the side arms of the element.

It is noted that our design has been applied in a radar system with more than 6000 elements, recently, exhibiting a manufacturing yield over 98%, which validates the reliability of the integrated approach.

## 4. Experimental Results

**Figure 9:**
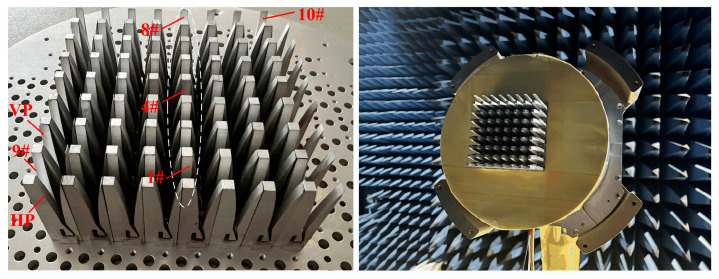
Photograph of the fabricated 8 × 8 Vivaldi array and test environment.

**Figure 10:**
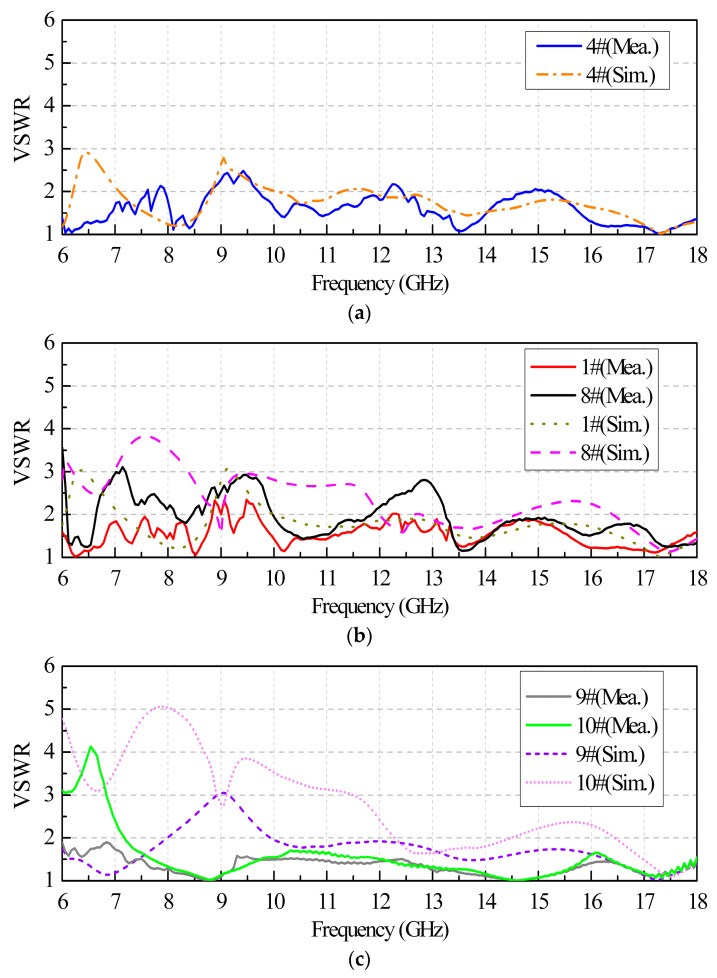
Simulated and measured VSWRs of selected elements in the 8 × 8 array. (**a**) Central element. (**b**) Edge element. (**c**) Corner element.

To demonstrate the above investigation, a prototype of an 8 × 8-element planar array is fabricated, shown in Figure 9. During machining, the tolerance of the size *w* is limited to 0/−0.05 mm (acceptable range: 10.45 mm to 10.5 mm). As can be seen from Figure 9, a circular metal plate with a thickness of 4 mm is employed for holding all the elements together. Threaded-through holes for M2 screws are drilled on the plate for mounting the elements. The VSWRs of embedded elements are measured by the Agilent E5071B vector network analyzer. Aside from the port to be measured, the other ports are terminated with 50 Ω loads. The simulated and measured VSWR curves of the 1#, 4#, and 8# elements are shown in Figure 10. The measured VSWR of the central element is generally less than 2.5 across the entire operating band. The matching of the edge and corner elements deteriorates, and the measured VSWRs increase to 4.1 due to the loss-adjacent elements that play a coupling role.

Then, we built a phased array consisting of antenna elements, T/R modules, a Wilkinson power-divider network, and a beam controller. The array is connected with eight 8-channel T/R modules via RF connectors, using integrated phase shifters and SPI communication to control the phase of each element. The assembled phased array was characterized in the anechoic chamber for its radiation performance. For simplicity, only the E- and H-plane radiation patterns of the vertical polarization (VP) array are shown.

**Figure 11:**
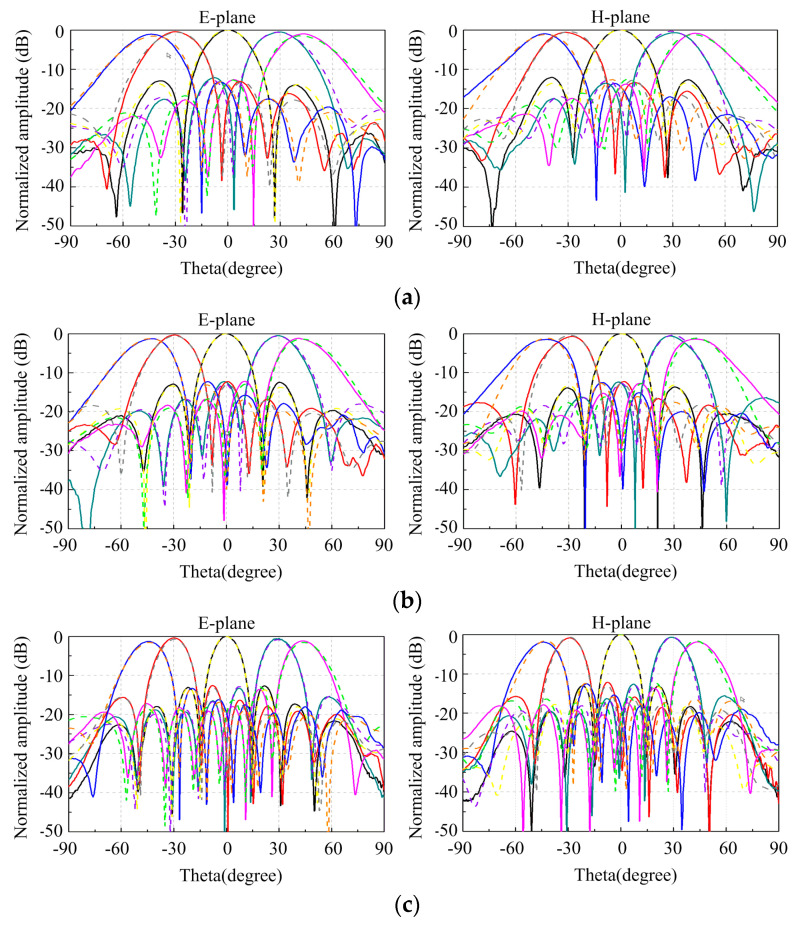

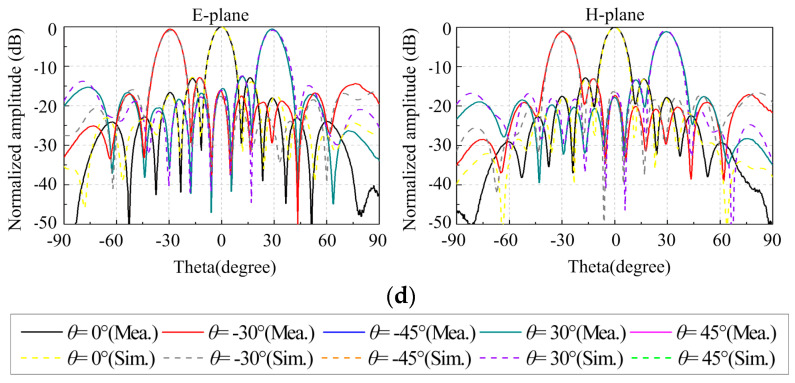
Simulated and measured normalized radiation patterns of the VP array in the E- and H-planes: (**a**) 6 GHz, (**b**) 10 GHz, (**c**) 14 GHz, and (**d**) 18 GHz.

**Figure 12:**
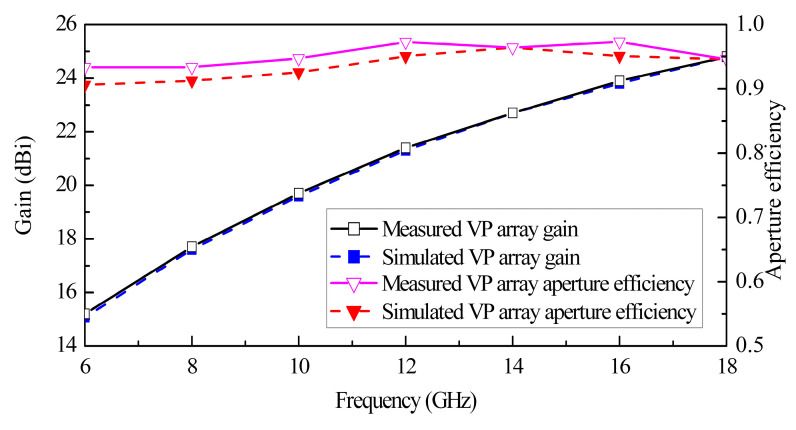
Simulated and measured gain and aperture efficiency results for the VP array.

The measured normalized scanning radiation patterns in the *xz*-plane and *yz*-plane at the frequencies of 6 GHz, 10 GHz, 14 GHz, and 18 GHz for the VP array are shown in Figure 11. It can be seen that the measured radiation patterns are almost in reasonable agreement with the simulated ones. No grating lobes appear when the array scans to 45° over the operating band. With the increase in the scanning angle, the array gain drops less than 1.8 dB in the *xz*-plane and 1.9 dB in the *yz*-plane within the entire band. The simulated and measured gain results of the array are presented in Figure 12. The gain exhibited by the array goes from 15.2 dBi at the lowest working frequency of 6 GHz up to 24.8 dBi at the highest frequency of 18 GHz, which is slightly higher than the simulated ones. This is attributed to the circular metal plate, which reduces the backward radiation and increases the effective aperture. The aperture efficiencies are also plotted in Figure 12. The measured aperture efficiencies are greater than 91% in the entire band, which is similar to the simulated results. As the number of elements increases, the aperture efficiency will further improve due to the decrease in the proportion of edge elements with poor VSWRs.

Above all, it is seen from the measurement results that the fabricated array can successfully provide an operating band from 6 GHz to 18 GHz without any singularities.

A comparison between the proposed metal Vivaldi array and some other similar arrays is shown in Table 2. However, in our design, the array is formed from independent elements, while all other studies use integrated processing methods, whether machining or additive manufacturing.

## 5. Conclusions

A dual-polarized metal Vivaldi array based on independent structural elements has been investigated and fabricated in this work, operating at 6–18 GHz and achieving a good scanning performance in the E- and H-planes. Compared with previous studies on metal Vivaldi arrays, the independent structural elements are more flexible for forming arrays. Absorbing material of specific sizes attached to the side arms of the element can eliminate the resonance caused by structural discontinuities and enhance the bandwidth effectively. Experiments were carried out to validate the design concept and method, showing good agreements between simulations and measurements. The gain of the array goes from 15.2 to 24.8 dBi in the operating band, and the aperture efficiencies are greater than 91%. In conclusion, the proposed approach to designing a metal Vivaldi element and array could be a good candidate for ultra-wideband system applications.

## Figures and Tables

**Table 1:** Element dimensions.

Parameter	Value (mm)	Parameter	Value (mm)
*h*	31.6	*h*_3_	1.0
*w*	10.5	*w*_3_	0.7
*h*_1_	5.0	*h*_4_	5.9
*w*_1_	1.4	*w*_4_	1.4
*h*_2_	2.3	*d*	3.0
*w*_2_	0.7		

**Table 2:** Comparison for the related metal Vivaldi arrays.

Ref.	Frequency (GHz)	Polarization	Manufacturing	Module Size
[16]	2–18	Dual	Machining	10 × 10
[18]	18–30	Dual	3D printing	4 × 4
[20]	26–40	Dual	Machining/3D printing	8 × 8
[21]	24.0–29.5	Single	3D printing	8 × 8
This work	6–18	Dual	Machining	1 × 1

## Data Availability

Data are contained within the article.

## References

[1] Li R., Li P., Rocca P., Salas Sánchez A.Á., Song L., Li X., Xu W., Fan Z. (2023). Design of Wideband High-Gain Patch Antenna Array for High-Temperature Applications. Sensors.

[2] Li Q., Nie C., Liu Z., Zhou X., Cheng X., Liang S., Yao Y. (2023). Circularly Polarized Ultra-Wideband Antenna for Uni-Traveling-Carrier Photodiode Terahertz Source. Sensors.

[3] Lu P., Liu Z., Lei E., Huang K., Song C. (2023). Superdirective Wideband Array of Circular Monopoles with Loaded Patches for Wireless Communications. Sensors.

[4] Ding G., Li P., Rocca P., Chang J., Xu W. (2022). Surrogate-Model-Based Interval Analysis of Spherical Conformal Array Antenna with Power Pattern Tolerance. Sensors.

[5] Li P., Liu W.G., Ren Z.M., Meng W.J., Song L.W. (2022). A High-Temperature and Frequency-Reconfigurable Multilayer Frequency Selective Surface Using Liquid Metal. IEEE Access.

[6] Gibson P.J. The Vivaldi aerial. Proceedings of the 9th European Microwave Conference.

[7] Schaubert D.H., Kasturi S., Boryssenko A.O., Elsallal W.M. Vivaldi antenna arrays for wide bandwidth and electronic scanning. Proceedings of the Second European Conference on Antennas and Propagation (EuCAP).

[8] Yao Y., Liu M., Chen W., Feng Z. (2010). Analysis and design of wideband widescan planar tapered slot antenna array. IET Microw. Antenna Propag..

[9] Langley J., Hall P., Newham P. (1996). Balanced antipodal Vivaldi antenna for wide bandwidth phased arrays. IEE Proc. Microw. Antennas Propag..

[10] Chio T.H., Schaubert D.H. (2010). Parameter Study and design of wide-band widescan dual-polarized tapered slot antenna arrays. IEEE Trans. Antennas Propag..

[11] Holter H., Chio T.H., Schaubert D.H. (2000). Experimental results of 144-element dual-polarized endfire tapered-slot phased arrays. IEEE Trans. Antennas Propag..

[12] Yngvesson K.S., Schaubert D.H., Korzeniowski T.L., Kollberg E.L., Thungren T., Johansson J.F. (1985). Endfire tapered slot antennas on dielectric substrates. IEEE Trans. Antennas Propag..

[13] Hood A.Z., Karacolak T., Topsakal E. (2008). A small antipodal Vivaldi antenna for ultrawide-band applications. IEEE Antennas Wirel. Propag. Lett..

[14] Pfeiffer C., Steffen T., Phillips G., Tomasic B. High power AESAs for 20–60 GHz with linear and circular polarizations. Proceedings of the IEEE International Symposium on Phased Array System & Technology (PAST).

[15] Kahkonen H., Ala-Laurinaho J., Viikari V. (2020). Dual-polarized Ka-band Vivaldi antenna array. IEEE Trans. Antennas Propag..

[16] Sapari L., Hout S., Chung J.-Y. (2022). Brain Implantable End-Fire Antenna with Enhanced Gain and Bandwidth. Sensors.

[17] Slimi M., Jmai B., Dinis H., Gharsallah A., Mendes P.M. (2022). Metamaterial Vivaldi Antenna Array for Breast Cancer Detection. Sensors.

[18] Kindt R.W., Pickles W.R. (2010). Ultrawideband all-metal flared-notch array radiator. IEEE Trans. Antennas Propag..

[19] Kindt R.W., Logan J.T., Elsallal M.W. Machined Metal FUSE Array Apertures. Proceedings of the IEEE International Symposium on Phased Array System & Technology (PAST).

[20] Kindt R.W., Logan J.T. (2020). Dual-polarized metal-flare sliced notch antenna array. IEEE Trans. Antennas Propag..

[21] Yan J., Gogineni S., Camps-Raga B., Brozena J. (2022). A Dual-Polarized 2–18-GHz Vivaldi Array for Airborne Radar Measurements of Snow. IEEE Trans. Antennas Propag..

[22] Kedar A. (2022). Dielectric Free Wide Scan UWB Low Cross-Pol Metallic Vivaldi Antenna for Active Phased Array Radars. IETE J. Res..

[23] Kahkonen H., Ala-Laurinaho J., Viikari V. (2022). A Modular Dual-Polarized Ka-Band Vivaldi Antenna Array. IEEE Access.

[24] Pfeiffer C., Massman J., Steffen T. (2021). 3-D Printed Metallic Dual-Polarized Vivaldi Arrays on Square and Triangular Lattices. IEEE Trans. Antennas Propag..

[25] Kahkonen H., Proper S., Ala-Laurinaho J., Viikari V. (2022). Comparison of Additively Manufactured and Machined Antenna Array Performance at Ka-Band. IEEE Antennas Wirel. Propag. Lett..

[26] Haarla J., Ala-Laurinaho J., Viikari V. (2023). Scalable 3-D-Printable Antenna Array With Liquid Cooling for 28 GHz. IEEE Trans. Antennas Propag..

[27] Li Y., Li X., Tao J., Liu C. Modular All Metal Vivaldi Antenna With 9:1 Bandwidth. Proceedings of the 2022 Cross Strait Radio Science & Wireless Technology Conference (CSRSWTC).

[28] Mailloux R. (2001). Phased Array Antenna Handbook.

[29] Harrinton R.F. (1961). Harmonic Electromagnetic Fields, Electrical and Electronic Engineering Series.

